# Prediction of early falls using adherence and balance assessments in a convalescent rehabilitation ward

**DOI:** 10.20407/fmj.2022-037

**Published:** 2023-11-29

**Authors:** Toshio Teranishi, Megumi Suzuki, Masayuki Yamada, Akiko Maeda, Motomi Yokota, Naoki Itoh, Masanori Tanimoto, Aiko Osawa, Izumi Kondo

**Affiliations:** 1 Faculty of Rehabilitation, Fujita Health University, School of Health Sciences, Toyoake, Aichi, Japan; 2 National Center for Geriatrics and Gerontology, Department of Rehabilitation Medicine, Obu, Aichi, Japan

**Keywords:** Convalescent rehabilitation ward, Fall prediction, Adherence assessment

## Abstract

**Objectives:**

To predict falls by adding an adherence assessment to a static balance ability assessment, and to evaluate fall prediction accuracy.

**Methods:**

This study included 416 patients who were admitted to a 45-bed convalescent rehabilitation ward over a 2-year period. The patients were assessed at the time of admission using the Standing Test for Imbalance and Disequilibrium (SIDE) and three additional, newly developed adherence items. Patients were divided into two groups: a group that experienced falls (fall group) and a group that did not experience falls (non-fall group) within 14 days of admission. The sensitivity and specificity of the assessment items for predicting falls were calculated.

**Results:**

Sensitivity was 0.86 and specificity was 0.42 when the cutoff was between SIDE levels 0–2a and 2b–4. Combining balance assessment using the SIDE with the memory and instruction adherence items improved fall prediction accuracy such that the sensitivity was 0.75 and the specificity was 0.64.

**Conclusions:**

Our analysis suggested that adherence assessment can improve fall risk prediction accuracy.

## Introduction

Falls are among the most common incidents in hospitals,^1–4^ and several fall risk assessment methods have thus been developed and are used in practice.^5–10^

The St. Thomas Risk Assessment Tool,^5^ Morse Fall Scale,^6^ and Hendrich Fall Risk Assessment^7^ are typical fall risk assessment measures used in hospital wards. These instruments are used for fall risk assessment in hospital wards in both Europe and the United States, whereas in Japan the Fall Risk Assessment Sheet^8^ recommended by the Japan Medical Association, the Assessment Sheet^9^ suggested by the Japanese Nursing Association, and the Fall Risk Assessment Sheet^10^ for Stroke Patients endorsed by the Medical Safety Committee of the Convalescent Rehabilitation Ward Association are the most frequently used instruments. These assessment measures estimate fall risk by summing the scores for weighted items related to fall risk, such as history of falls, mobility impairment, use of medication, frequent urination, and incontinence.

**Figure 1:**
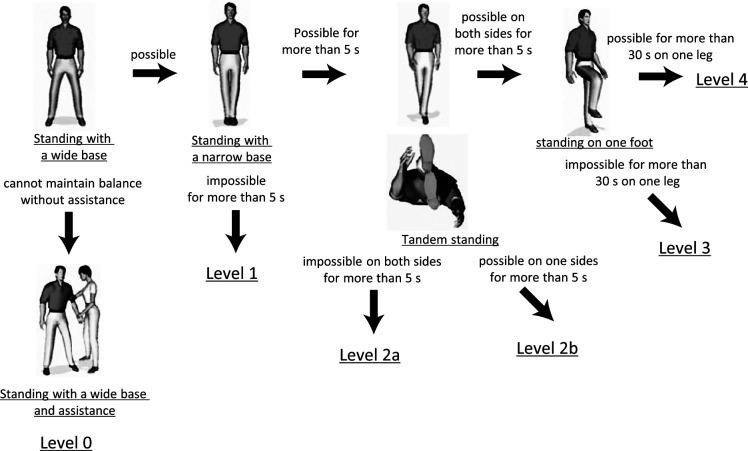
Flow chart showing the process used to determine the Standing Test for Imbalance and Disequilibrium (SIDE) levels. The levels are arranged in order of difficulty; more levels should not be included once a subject loses balance at a certain level and requires assistance. As the level of difficulty in the test increases, the risk of falling increases. Level 0: A standing position with a wide base cannot be maintained without assistance. Support provided by grasping something or being assisted by a caregiver is always required to maintain a standing position. Level 1: A standing position with a wide base can be maintained without assistance, but standing position with a narrow base cannot be maintained for more than 5 s. Balance is lost in a standing position with a narrow base: bring the leg close together such that the feet are in contact with each other medially at both the heel and forefoot. Level 2a: A standing position with a narrow base can be maintained for more than 5 s, but a tandem standing position cannot be maintained for more than 5 s with either leg position. The tandem standing position involves standing with the heel of one foot placed at the toe of the other foot, in a straight line (either foot may be in front). Level 2b: A tandem standing position can be maintained for more than 5 s with one but not the other leg in the leading position. Level 3: A tandem standing position can be maintained for more than 5 s with either leg in front, but standing on one leg is difficult to maintain for more than 30 s with either leg. Level 4: A position of standing on one leg can be maintained for more than 30 s with either leg.

Falls occur when patients perform activities or tasks that exceed their ability to maintain balance. In most cases, if the patient can maintain balance sufficiently, a trip or slip during an activity will not result in a fall. Thus, the ability to maintain good balance is the most important factor for avoiding falls. Therefore, we developed the Standing Test for Imbalance and Disequilibrium (SIDE), a simple test of a person’s ability to maintain static standing balance (Figure 1). After verifying the reproducibility and validity of the SIDE, we examined the ability to maintain balance of people who fell and the possibility of using the SIDE to predict falls.^11^

Falls in convalescent rehabilitation wards mostly occur early after admission.^12,13^ This is because the patient is unaccustomed to the new environment and the changes in physical abilities caused by their illness. Furthermore, the medical staff may not have a sufficient understanding of the patient’s fall risk. In addition, patients in rehabilitation wards engage in more activities compared with those in acute wards.

A study that applied the SIDE to patients who fell within 14 days after admission to a convalescent rehabilitation ward reported that the incidence of falls, although high overall, was lower in patients with good balance.^14^

Although patients at risk of falling can be identified using the SIDE, false positives can occur. Thus, it is necessary to develop a secondary detection tool to improve the sensitivity, specificity, and accuracy of fall predictions.

In this study, parameters not assessed by the SIDE, such as self-perceived ability to maintain balance and impulsivity, were covered by a newly devised assessment encompassing personality, memory, and adherence to instructions. The purpose of the study was to examine the utility of combining the SIDE with a newly developed adherence assessment for identifying people who may experience a fall within 14 days after admission to a convalescent rehabilitation ward.

## Methods

This study had two stages. In the first stage, an adherence assessment was developed. In the second stage, adherence assessment and SIDE data (Figure 1) were obtained for all participants. In addition, information on the time of fall was obtained from each faller.

### Development of the adherence assessment

**Figure 2:**
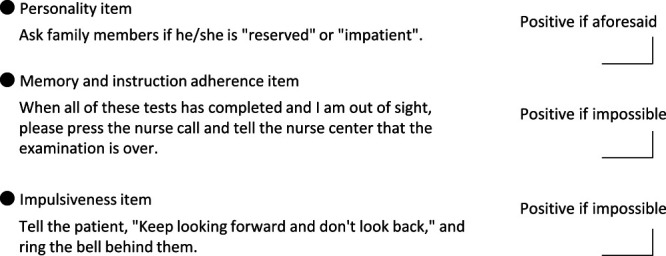
Adherence items

The adherence assessment was developed to identify people who are unable to stop themselves from performing dangerous acts when their movement is restricted. Seven experienced professionals (one physiatrist, two physical therapists, two occupational therapists, and two nurses) and one coordinator used the nominal group technique and devised assessment items and methods. After lengthy discussions, assessment items were rated on Likert scales and classified as personality, memory and instruction adherence, or impulsiveness items. Items for which classification agreement was low were the subject of further discussion (Figure 2). Regarding personality items, on the basis of interviews with the patient’s family, the patient was characterized as “reserved” or “impatient.” Memory and instruction adherence was assessed by asking the patient to inform the nursing center when the test was over; participants were classified according to their ability to do this. Finally, patients were classified as impulsive if they looked back in response to the following instruction: “Keep looking forward and don’t look back.”

### Acquisition of adherence assessment and SIDE data

**Figure 3:**
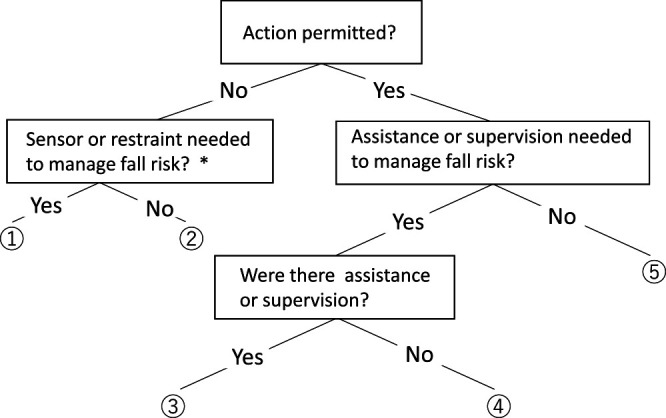
Classification of fall cases according to the patient management method ① to ⑤ show the classification according to the management method. ① with sensor or restraints; ② without sensor or restraints; ③ supervised or assisted; ④ nurse left during supervising/assistance; ⑤ patient permitted to perform action unsupervised alone. *Physical restraints were applied only after evaluating the need therefor and obtaining consent from the patient.

Adherence assessment and SIDE data were obtained on the day of admission to the convalescent rehabilitation ward for all patients. Falls occurring within 14 days after admission were classified according to the patient management method (Figure 3).^12^ First, the patient’s action at the time of the fall was classified according to whether it was permitted or not. Permitted actions were further categorized according to the presence versus absence (labeled as ① and ②, respectively) of sensors or restraints. Moreover, actions requiring and not requiring assistance or supervision were labeled as ③ and ④, respectively. Finally, actions that patients were permitted to perform unassisted were labeled as ⑤.

Falls were defined as “when a part other than the sole of the feet touches the floor or ground against one’s own will”.^15^

### Data analysis

The data are presented as proportions, mean, or median values as appropriate. The participants were divided into fall and non-fall groups depending on whether they experienced a fall in the 14 days post admission. The fall and non-fall groups were compared in terms of SIDE performance and the personality, memory and instruction adherence, and impulsiveness measures using Fisher’s exact test. The Youden index was used to maximize the sensitivity and specificity for predicting falls within 14 days of admission of assessment measures that showed differences between the two groups. Furthermore, the relationship between the classification of fall cases according to the patient management method and the adherence assessment results (“positive” or “negative”) was examined. We used Prism 5 software (GraphPad Software Inc., San Diego, CA, USA) to perform the analyses and the significance level was set to 5%.

This study was approved by the Ethics and Conflict of Interest Committee (Approval No. 792) of the National Center for Geriatrics and Gerontology and complied with the Declaration of Helsinki. The participants were given the option to opt out of the study if they wished and were assured that this would not affect the services provided by the ward.

## Results

This study included all 416 patients admitted to a 45-bed convalescent rehabilitation ward between April 1, 2015, and March 31, 2017. Table 1 shows the attributes of the participants. The participants comprised 416 patients (154 males and 262 females) with a mean (standard deviation) age of 77.9 (9.6) years (range: 38–102 years). The underlying pathologies/histories of the patients included femoral neck fracture (n=65), cerebral hemorrhage (n=49), cerebral infarction (n=98), spinal cord injury (n=8), vertebral compression fracture (n=46), and other (n=150). The mean (standard deviation) Functional Independence Measure (FIM) motor and cognitive subscale scores on admission were 49.7 (19.5) and 25.5 (7.5) points, respectively. The FIM^16^ was developed as a measure of independence in activities of daily living for patients. The FIM consists of 18 items (13 and 5 related to the motor and cognitive domains, respectively), and each item is rated on a 7-point (range: 1–7) ordinal scale. Total scores range from 18 to 126 points.

Thirty-eight patients experienced a fall within 14 days of admission. Table 2 compares the fall and non-fall groups in terms of SIDE performance. There was a significant difference in the number of fall cases between SIDE levels 0–2a (n=31) and 2b-4 (n=5; p<0.05), and between SIDE levels 0–2b (n=35) and 3–4 (n=1; p<0.05).

The Youden index was 0.28 for the comparison between SIDE levels 0–2a and 2b–4 (sensitivity=0.86, specificity=0.42) and 0.25 for the comparison between SIDE levels 0–2b and 3–4.

Table 3 is a contingency table for the three assessment items according to the presence or absence of falls. There was no significant difference between the fall and non-fall groups in the personality or impulsiveness item results, but there was a significant group difference for the memory and instruction adherence item results (p<0.05).

The memory and instruction adherence item and SIDE data were used for fall prediction. In total, 390 participants were included in the analysis after excluding 18 with missing SIDE data and/or missing memory and instruction adherence item data (n=14; 6 participants had missing data for both metrics). After excluding a further 103 patients of SIDE level 3–4, 287 patients were divided into fall and non-fall groups, and each of those groups was further divided into positive and negative subgroups according to the instruction adherence item result. The 103 SIDE level 3–4 cases omitted in the previous step were then added to the negative subgroup, and sensitivity, specificity, and Youden index values were calculated for the 390 cases; the values were 0.75, 0.64, and 0.39, respectively). Table 4 summarizes the sensitivity and specificity for SIDE performance only (cutoff between SIDE levels 2a and 2b), the memory and instruction adherence item result only, and both metrics. Patients with a SIDE level of 0–2b and a positive result on the memory and instruction adherence item were considered to be at higher risk of falling.

Table 5 shows the relationship between the fall case classification according to the patient management method and the memory and instruction adherence item positivity rate. The positivity rate was 87.5% (14 cases) for falls that occurred when the patient was restrained but slipped through the restraints, 100.0% (7 cases) for falls occurring under staff supervision, and 57.1% (4 cases) for falls that occurred while the patient was performing a permitted action alone or was performing an action that required supervision during which the supervisor took his/her eyes off the patient.

## Discussion

In this study, we assessed balance in all patients presenting to a convalescent rehabilitation ward within a 2-year period using the SIDE and newly developed adherence assessment measures, with the goal of predicting falls. We enrolled representative patients with indications for rehabilitation, such as femoral neck fractures, stroke, and vertebral compression fractures. Patients with a high SIDE level, i.e., those with a high ability to maintain balance, are less likely to fall. Therefore, after excluding patients with high SIDE levels, our adherence assessment measures may predict fall risk more accurately. However, patients for whom weight bearing on the lower extremities is prohibited, such as those who have suffered a fracture, cannot be accurately identified using tests require bilateral lower extremity weight bearing (SIDE level 0–3).

The risk of falls in people with good balance is low.^14^ In this study, there were five fall cases with a SIDE level of 2b or higher and one case with a SIDE level of 3 or higher. However, not all people with poor balance will experience a fall, such that there is a need to identify potential fallers among those with poor balance. A fall may occur when a person performs an action that exceeds their ability to maintain balance. Even if a patient has poor balance, they are unlikely to fall if the activity is not among those that compromises their ability to maintain balance. A previous logistic regression analysis of scores on FIM items associated with the occurrence of falls found that low problem-solving ability increased the likelihood of falls;^14^ such items include “irrelevant actions” and “performing dangerous actions”.^16^ We attempted to improve fall prediction accuracy by devising adherence assessment measures and combining them with the SIDE for assessment of the ability to maintain balance.

The adherence assessment developed in this study consisted of personality-, memory and instruction adherence-, and impulsiveness-related items. A significant difference was observed between the fall and non-fall groups only for the memory and instruction adherence item results. Finally, after identifying individuals with good balance using the SIDE, those with poor balance were assessed on the basis of the memory and instruction adherence item results. For 390 cases, on the basis of the SIDE and memory and instruction adherence item results, the Youden Index was 0.39, the sensitivity was 0.75, and the specificity was 0.64.

In this study, falls caused by patients lacking sensors or restraints when performing difficult actions, and falls occurring during the performance of actions permitted without supervision or assistance, were associated with a lower positivity rate (57.1%) for the memory and instruction adherence item than those associated with falls occurring when using sensors or restraints (87.5%) or while under supervision (100%). The high positivity rates in the latter two circumstances show that falls can occur even under supervision. Therefore, the measures taken and techniques used by medical staff to prevent falls require further consideration. The positivity rate associated with falls occurring in the absence of sensors or restraints, and that associated with falls that patients were permitted to perform unassisted, were almost equal, and it is not clear whether the cause of the falls in these cases was poor balance, a decline in cognitive function, or both.

There were some limitations to this study. First it was a single-center study. Furthermore, there is scope for improvement of the personality- and impulsiveness-related assessment items. For example, the impulsiveness assessment item such as “Keep looking forward and don’t look back” when the bell rings may not elicit correct response. Whether a task induces impulsiveness depends on the patient’s motivation; thus, it can be difficult to assess this behavioral trait. For the personality assessment, an interview survey was conducted enquiring about the participants’ health conditions before illness onset. It may be necessary to conduct a behavioral assessment at the same time as the adherence assessment.

## Figures and Tables

**Table1:** The backgrounds of the 416 patients in this study

Age (years old)	77.9±9.6 range 38–102
Gender (cases)	Male 154, Female 262
Diagnosis (cases)	Femoral neck fracture 65, Cerebral hemorrhage 49, Cerebral infarction 98, Spinal cord injury 8, Vertebral compression fracture 46, Others 150
FIM score at the admission (points)	FIM motor 49.7±19.5, FIM cognitive 25.5±7.5

**Table2:** Comparison of fall group and no fall group by SIDE level (416 cases)

	SIDE*	
	fall group (38 cases)	no fall group (378 cases)
level 0	31.6% (12)	19.1% (72)
level 1	13.2% (5)	11.9% (45)
level 2a	36.8% (14)	24.6% (93)
level 2b	10.5% (4)	13.2% (50)
level 3	2.6% (1)	20.4% (77)
level 4	0.0% (0)	6.6% (25)
not testable	5.3% (2)	4.2% (16)

* SIDE level 0–2a/level 2b–4 (p<0.05), SIDE level 0–2b/level 3–4 (p<0.05)

**Table3:** Three adherence items and split table of whether or not there is a fall (416 cases)

	Personality item (reserved or impatient) n.s.			Memory and instruction adherence item p<0.05			Impulsiveness item n.s.	
	faller (38 cases)	non faller (378 cases)		faller (38 cases)	non faller(378 cases)		faller (38 cases)	non faller (378 cases)
positive	60.5% (23)	69.8% (264)		76.3% (29)	42.1% (160)		5.3% (2)	4.2% (16)
negative	39.5% (15)	29.1% (110)		23.7% (9)	54.0% (204)		94.7% (36)	91.3% (345)
missing	0.0% (0)	1.1% (4)		0.0% (0)	3.7% (14)		0.0% (0)	4.5% (17)

**Table4:** Comparison of predictions based on “SIDE”, “Memory and instruction adherence” and “combination of both”

	sensitivity	specificity	Youden Index
SIDE (cut-off value 2a/2b) n=398	0.86	0.42	0.28
Memory and instruction adherence （positive/negative）n=402	0.76	0.56	0.32
Combining SIDE with Memory and instruction adherence（positive/negative）n=390	0.75	0.64	0.39

**Table5:** Relationship between the classification of fall cases according to the management method and the memory and instruction item positive rate

	falls (cases)	Memory and Instructional Adherence positeive (cases)	Percentage (%)
① With sensor or restraint	16	14	87.5
② Without sensor or restraint	7	4	57.1
③ With supervised or assisted	7	7	100.0
④ Left the scene during monitoring assistance	1	0	0.0
⑤ Permit do it alone	7	4	57.1
total	38	29	76.3
